# A Comprehensive Analysis of Argonaute-CLIP Data Identifies Novel, Conserved and Species-Specific Targets of miR-21 in Human Liver and Hepatocellular Carcinoma

**DOI:** 10.3390/ijms19030851

**Published:** 2018-03-14

**Authors:** Aaron Balasingam Koenig, Juan Martín Barajas, María Jose Guerrero, Kalpana Ghoshal

**Affiliations:** 1 Department of Pathology, Comprehensive Cancer Center, The Ohio State University, Columbus, OH 43210, USA; aaron.koenig@osumc.edu (A.B.K.); barajas.10@osu.edu (J.M.B.); guerrero.41@buckeyemail.osu.edu (M.J.G.); 2 Medical Student Research Program, The Ohio State University College of Medicine, Columbus, OH 43210, USA

**Keywords:** miRNA, miR-21, liver cancer, HCC

## Abstract

MicroRNAs are ~22 nucleotide RNAs that regulate gene expression at the post-transcriptional level by binding messenger RNA transcripts. miR-21 is described as an oncomiR whose steady-state levels are commonly increased in many malignancies, including hepatocellular carcinoma (HCC). Methods known as cross-linking and immunoprecipitation of RNA followed by sequencing (CLIP-seq) have enabled transcriptome-wide identification of miRNA interactomes. In our study, we use a publicly available Argonaute-CLIP dataset (GSE97061), which contains nine HCC cases with matched benign livers, to characterize the miR-21 interactome in HCC. Argonaute-CLIP identified 580 miR-21 bound target sites on coding transcripts, of which 332 were located in the coding sequences, 214 in the 3′-untranslated region, and 34 in the 5′-untranslated region, introns, or downstream sequences. We compared the expression of miR-21 targets in 377 patients with liver cancer from the data generated by The Cancer Genome Atlas (TCGA) and found that mRNA levels of 402 miR-21 targets are altered in HCC. Expression of three novel predicted miR-21 targets (CAMSAP1, DDX1 and MARCKSL1) correlated with HCC patient survival. Analysis of RNA-seq data from SK-Hep1 cells treated with a miR-21 antisense oligonucleotide (GSE65892) identified RMND5A, an E3 ubiquitin ligase, as a strong miR-21 candidate target. Collectively, our analysis identified novel miR-21 targets that are likely to play a causal role in hepatocarcinogenesis.

## 1. Introduction

Liver cancer is the second leading cause of cancer-related death in the world [1]. Hepatocellular carcinoma (HCC) is a tumor of epithelial origin that typically arises in patients with underlying liver disease [2]. Risk factors for HCC are chronic infection with hepatitis B or C viruses, diabetes, alcohol and nonalcoholic steatohepatitis, exposure to carcinogens such as aflatoxin B_1_ and genetic diseases such as hereditary hemochromatosis, α_1_-anti-trypsin deficiency or tyrosinemia [2]. Chronic liver inflammation, oxidative stress, and hypoxia associated with the underlying disease lead to the accumulation of oncogenic driver mutations eventually progressing to HCC [3,4,5].

MicroRNAs are small RNAs (18 to 25 nucleotides) that regulate gene expression at the post-transcriptional level typically by imperfectly base-pairing with the 3′ untranslated region (3′UTR) of mRNA in association with the RNA-induced silencing complex (RISC) [6]. This interaction represses overall protein levels by inducing mRNA destabilization or inhibiting its translation [6]. MicroRNA-21 (miR-21) is a conserved and ubiquitously expressed miRNA coded from the 3′UTR of *TMEM49* gene on chromosome 17 in humans [7]. However, transcription of primary miR-21 is regulated by a promoter distinct from that of *TMEM49*. Overexpression of miR-21 contributes to disease progression in many hematologic malignancies and solid tumors [8,9,10]. In primary HCC tissues, upregulation of miR-21 expression correlates with tumor grade [11]. In diseased liver, inflammatory cytokines stimulate transcription of *MIR21* through transcription factors STAT3 and AP-1 [12,13,14]. miR-21, in turn, suppresses the expression of tumor suppressors such as phosphatase and tensin homolog (PTEN) [15], programmed cell death protein 4 (PDCD4) [16,17,18], reversion-inducing-cysteine-rich protein with kazal motifs (RECK), and metalloproteinase inhibitor 3 (TIMP3) by direct targeting [19].

miR-21 has been shown to be upregulated in primary human HCCs [15]. However, identity of miR-21 targets and their regulation in the transcriptome of tumors and host livers have not been described. Recently, our laboratory performed high-throughput sequencing of RNA isolated by cross-linking and immunoprecipitation of Argonaute (Ago-CLIP) [20] to identify microRNAs and their target mRNAs in mouse livers and human livers with their matching HCC tumor. We recently reported significant enrichment of miR-21 and its targetome in HCCs compared to the benign livers [21]. Here, we expand on our previous findings and perform a transcriptome-wide characterization of the miR-21 interactome in primary human HCCs and benign livers using data from our previous Ago-CLIP analysis (GSE97061) [21]. Many novel targets were identified whose functional roles in hepatocarcinogenesis require further study.

## 2. Results

### 2.1. miR-21 Binds Broadly within Liver and HCC Transcriptomes

**Figure 1:**
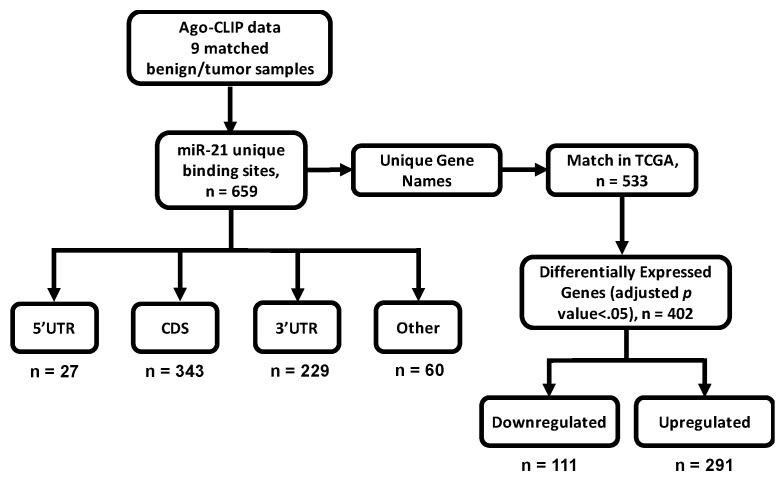
Workflow for the selection and analysis of microRNA-21 (miR-21) targets. Unique miR-21 targets (*n* = 659) were identified using high-throughput sequencing of RNA isolated by cross-linking and immunoprecipitation of Argonaute (Ago-CLIP) data generated from matching benign liver and tumor tissues isolated from nine hepatocellular carcinoma (HCC) patients (GSE97061) [21]. Targets were sorted based on annotation of target loci: 5′ untranslated region [5′UTR], coding sequence [CDS], 3′ untranslated region [3′UTR], or other. Other groups included were transposable elements, introns, etc. Unique targets were compared to RNA-seq data generated by The Cancer Genome Atlas (TCGA) after log_2_ transformation and differential expression analysis between benign tissue and tumor tissue using the limma package in R [22]. Significance for differentially expressed genes was defined as having an adjusted *p*-value < 0.05 (*n* = 402).

**Figure 2:**
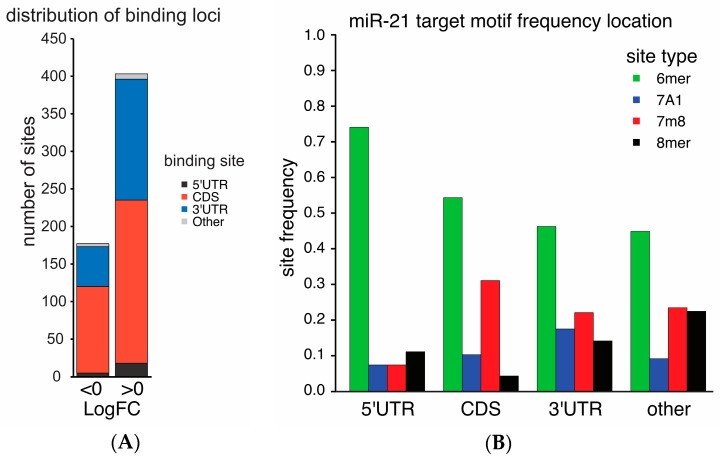
Binding sites for miR-21 are most commonly in the CDS and 3′UTR of mRNA with variation in binding motif frequency. miR-21 binding sites identified by Ago-CLIP were classified by the sign of their log-fold enrichment in HCC and by the location of miR-21 binding on the target transcript: 5′ untranslated region, CDS, 3′UTR or other (**A**). The binding sites of miR-21 were classified by the frequency of the target sequence motif (6mer, 7A1, 7m8, 8mer) at each annotated location (**B**). The distribution of sequence motifs varied between the CDS and the 3′UTR (*p* = 2.1 × 10^−6^).

Recent Ago-CLIP analysis (GSE97061) of tumor and benign liver tissues from nine HCC patients identified miR-21 along with 659 of its targets associated with Argonaute. We classified 581 miR-21 binding sites in gene targets based on their enrichment in HCC and normal liver (Figure 1). Overall, 69.5% of miR-21 targets were enriched in HCCs. The majority of miR-21 binding sites enriched in HCC or in liver were located in either the coding sequence (CDS) (218/404 sites with logFC > 0 in HCC, 115/177 sites with logFC < 0 in HCC) or 3′UTR (161/404 sites with logFC > 0 in HCC, 53/177 sites with logFC < 0 in HCC, Figure 2A).

MicroRNA binding affinity varies by target motifs among 6mer, 7merA1, 7merM8, and 8mer sites by the degree of base pairing [23]. The distribution of miR-21 target motifs varied between coding sequences (CDS) and the 3′UTR (*p* = 2.1 × 10^−6^), with the CDS having a paucity of 8mer binding sites compared to its binding sites in the 3′UTR (Figure 2B). Overall, 6mer sites were the most common motif.

### 2.2. Conservation of miR-21 Targets between Mice and Humans

**Figure 3:**
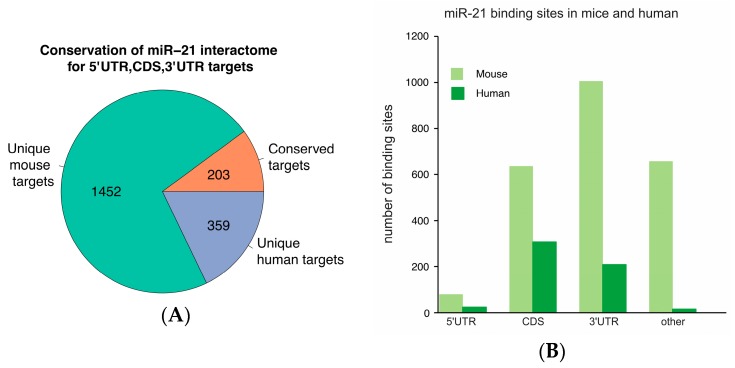
A low number of miR-21 sites are conserved among mice and humans. Targets in human livers or HCCs that precipitated miR-21 in Ago-CLIP (GSE97061) were compared to homolog gene identifiers from Ago-CLIP in mouse liver (GSE97058). Targets contained at least one binding site for miR-21 in the 5′UTR, CDS or 3′UTR (**A**). The number of binding sites at different locations in the mouse and human transcriptomes was compared (**B**).

To determine the conservation of miR-21 binding sites, we compared gene identifiers from mouse liver transcripts identified by Ago-CLIP that interact with miR-21 (GSE97058) with human liver and HCC Ago-CLIP data. Among 562 genes with miR-21 binding sites in the 5′ untranslated region (5′UTR), CDS or 3′UTR, 359 were unique to humans (Figure 3A). miR-21 binding sites in mouse livers were predominantly located in the 3′UTR whereas those in human livers or HCCs were more frequently found in the CDS (Figure 3B). An additional 1452 targets were identified in mouse livers (Supplementary Materials).

### 2.3. Certain Transcripts in the miR-21 Interactome Are Less Abundant in HCC

Matching of gene identifiers to RNA-seq data from 377 HCC patients and 59 normal livers in The Cancer Genome Atlas (TCGA) produced 533 gene matches with RNA-seq expression data. Of 402 miR-21 targets with significant change in expression in HCC, only 111 genes (27.6%) were downregulated, while 291 (72.4%) were upregulated (Supplementary Materials). Genes in the miR-21 interactome that were downregulated in HCC include *SPRY2* and *MAP2K3* that were previously validated as miR-21 targets (Table 1) [24,25,26]. Notably, several genes interacting with miR-21 were upregulated in HCC (Table 2).

### 2.4. Depletion of the miR-21 Interactome in Argonaute Complexes in HCC Is Associated with Repression

**Figure 4:**
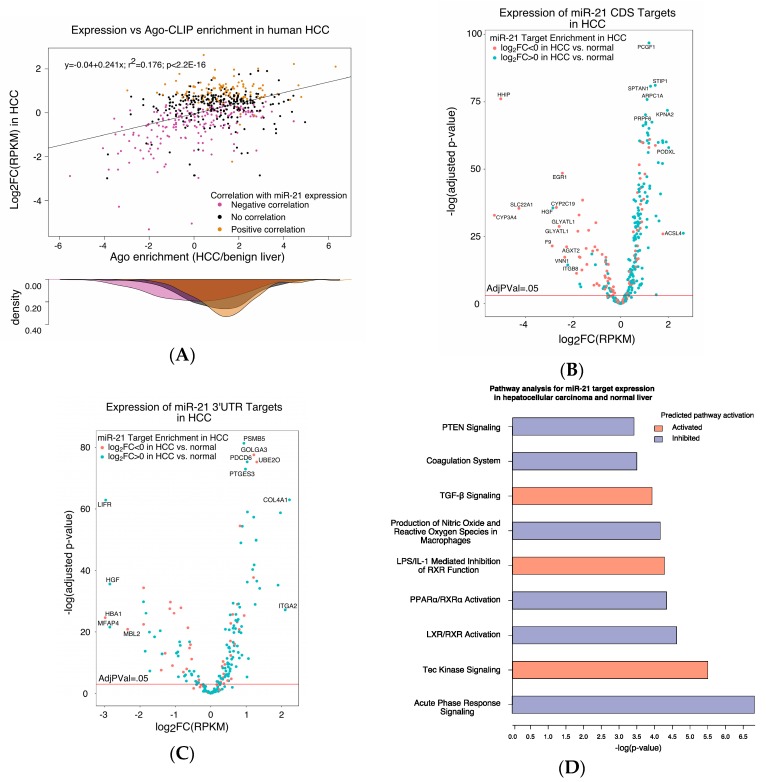
The expression data of the miR-21 interactome in tumor (*n* = 377) and matched normal tissue (*n* = 59) in patients with liver cancer were extracted from TCGA and compared to the tumor enrichment score determined by Ago-CLIP (**A**). Log_2_-fold enrichment of miR-21 targets by Ago-CLIP in HCC positively correlated with log_2_-fold expression change in tumor (*p* < 2.2 × 10^−16^). Genes whose expression in tumors negatively correlated with miR-21 expression were less enriched in HCC by Ago-CLIP than those with positive correlation (*p* < 2.2 × 10^−16^). Among miR-21 CDS targets, 64 genes were significantly downregulated and 173 genes were significantly upregulated (**B**). Among miR-21 3′UTR targets, 48 genes were significantly downregulated and 173 genes were significantly upregulated (**C**). Expression data for miR-21 targets in HCC were subjected to Ingenuity Pathway Analysis (IPA). Pathways predicted to be activated or inhibited by the miR-21 targetome are shown (**D**). Pathway significance was defined as having an adjusted *p*-value < 0.0005.

We then considered the outcome of miR-21 binding to the HCC transcriptome. We compared the Argonaute enrichment of miR-21 binding partners in HCC relative to normal tissue to their mRNA levels in HCC relative to normal tissue. The target enrichment score for Ago binding in HCC positively correlated with overexpression of the target in HCC relative to liver (*p* < 2.2 × 10^−16^). In HCC, transcripts in the miR-21 interactome whose abundance in tumors directly correlated with miR-21 expression were more enriched with Argonaute than transcripts whose expression inversely correlated with miR-21 expression (*p* < 2.2 × 10^−16^) (Figure 4A). Among the transcripts that interacted with miR-21, cytochrome 3A4, encoded by *CYP3A4*, and hedgehog inhibitory protein, encoded by *HHIP*, had an over 32-fold decrease in transcript abundance in HCC (Figure 4B,C). The expressions of miR-21 targets with binding sites outside of the CDS and 3′UTR are shown in Figure S1A,B, respectively.

### 2.5. The miR-21 Interactome Is Predicted to Regulate Tumor Metabolism

We subjected expression data of miR-21 targets from 377 HCC cases and 59 normal livers to Ingenuity Pathway Analysis (IPA). Signaling pathways predicted to be regulated in HCC by miR-21 targets include inhibition of acute phase response signaling, the liver X receptor/retinoid X receptor (LXR/RXR) pathway, the peroxisome proliferator-activated receptor alpha/retinoid X receptor alpha (PPARα/RXRα) pathway, phosphatase and tensin homolog (PTEN) signaling, and upregulation of Tec kinase signaling (Adjusted *p*-value < 0.0005, Figure 4D). The acute phase response is a systemic reaction to infection, neoplasm, tissue damage, or in inflammatory disorders that is associated with a change in hepatic metabolism [27]. Both PPARα/RXRα and LXR/RXR are ligand-activated transcription factors involved in regulation of liver metabolism, including fatty acid metabolism [28]. PTEN modulates cell cycle progression and cell survival by dephosphorylating phosphatidylinositol (3,4,5)-triphosphate and acting as a negative regulator of Akt signaling [15]. Tec kinases are non-receptor tyrosine kinases that regulate the development and activation of lymphoid and myeloid cells and play a role in liver regeneration [29,30,31].

### 2.6. An Unbiased Approach Identified RMND5A as a Potential miR-21 Target

**Figure 5:**
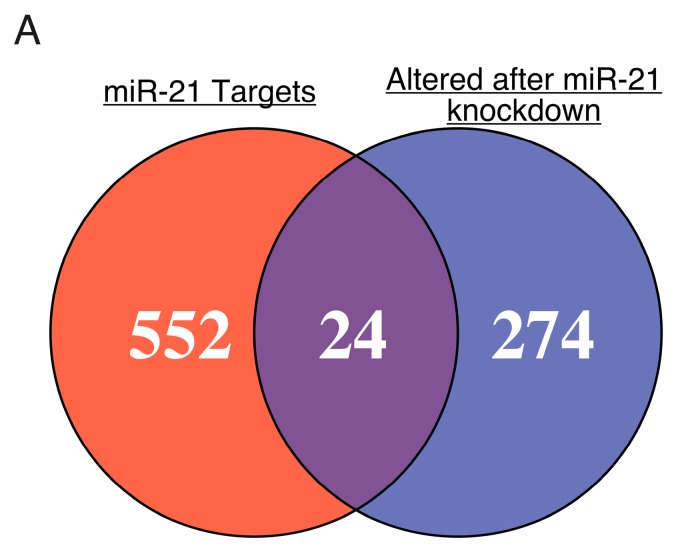
Gene identifiers from transcripts in association with miR-21 identified by Argonaute-CLIP were matched to genes dysregulated in SK-Hep-1 cells after treatment with anti-miR-21 (GSE65892) [33]. The 24 common genes were considered potential miR-21 targets.

We then focused on genes in the miR-21 interactome for further validation. We compared the miR-21 target list from Ago-CLIP with aberrantly expressed genes from published microarray data (GSE65892) in SK-Hep-1, a human endothelial cell line derived from the ascites fluid of a patient with liver adenocarcinoma [32], depleted of miR-21 with anti-sense oligo [33]. We identified 24 genes in the miR-21 interactome among 298 genes aberrantly expressed in SK-Hep-1 cells after anti-miR-21 treatment (Figure 5). The identity of these genes and their correlation with miR-21 expression in HCC are listed in Table 1. Upregulation of expression after anti-miR-21 treatment was observed in 16 of 24 dysregulated miR-21 targets [33]. Ago-CLIP analysis in mouse liver identified miR-21 binding sites in murine homologs of 10 of these 24 miR-21 targets (Table S1).

To investigate the functions of newly identified miR-21 targets, we queried the Ingenuity Knowledge Base for canonical pathway involvement and protein function (Table S2). Two genes identified by our analysis, *SMARCE1* and *TGFBR2*, shared function in glucocorticoid receptor signaling. B-cell receptor signaling and the downstream pathway of PI3K signaling were implicated by dysregulation of *PAG1* and *PLEKHA1*, respectively, in miR-21-depleted SK-Hep-1 cells.

Among potential miR-21 targets upregulated in miR-21-depleted SK-Hep-1 cells, abundance of *RMND5A,* transcript negatively correlated with miR-21 expression in HCC patients (Table 2). *RMND5A* bears a 7merA1 motif in the fifth CDS exon as its sole miR-21 binding site. *RMND5A*, also known as *CTLH*, encodes an E3 ubiquitin ligase in budding yeast [34].

### 2.7. Elements of the miR-21 Interactome Predict Survival in HCC

Next, we conducted a survival analysis on potential targets of miR-21 in 369 patients with HCC. Results are shown in Table 2. Among miR-21 candidate targets, higher expression of *CAMSAP2*, *DDX1*, and *MARCKSL1* predicted shorter survival. Expression of *MARCKSL1* positively correlated with miR-21 expression (Table 1). Expression data for *CAMSAP2* were not available in the TCGA RNASeq data set.

## 3. Discussion

Overexpression of miR-21 is a poor prognostic indicator in hepatocellular carcinoma and other solid tumors as well as hematologic malignancies [9,10,35]. Our analysis showed that the miR-21 interactome identified by Ago-CLIP modulates diverse pathways in HCC. The 562 gene transcripts that interact with miR-21 represent a substantial fraction of the liver transcriptome. We used these genes to investigate the functional consequences of miR-21 targeting in the setting of HCC.

We found that miR-21 targets whose expression varied inversely with miR-21 in HCC tended to be less bound to Argonaute. MicroRNAs can shorten the half-life of the messenger RNAs they bind to [36]. We expect that these transcripts in HCC are rapidly degraded following binding of miR-21, and would then be less enriched in Argonaute complex. It is likely that upregulation of miR-21 in HCC results in the repression of its targets. Surprisingly, we found that more genes in the miR-21 interactome were overexpressed than repressed in HCC. Both transcriptional and post-transcriptional mechanisms are involved in gene expression, and miR-21 represents one of the mechanisms that, in general, fine tune the expression of target genes [37]. However, we cannot rule out that miR-21 suppresses protein levels of some of these targets by blocking their translation [6]. 

Ago-CLIP analysis showed that a large number of functional miR-21 targets have binding sites in the CDS. MicroRNAs can mediate transcript depletion and/or translational inhibition by binding to CDS [38,39,40,41]. We found, however, that CDS miR-21 binding sites were less likely to be higher affinity sites, such as 8mer, than those in the 3′UTR. The extent of seed sequence complementarity may influence the ability of closely spaced microRNA binding sites to synergistically repress targets [23].

In our study, miR-21 was predicted to regulate multiple pathways in HCC, including the acute phase response, Tec kinase signaling, PTEN signaling, PPARα/RXRα signaling, and LXR/RXR signaling. Positive acute phase response reactants, including C-reactive protein, fibrinogen and complement components, are overexpressed in liver and released to systemic circulation, while synthesis of negative acute phase response reactants, such as transthyretin and albumin, is curtailed [27]. Ferrín et al. reported that acute phase reactants fibrinogen and the C4a fragment of complement component 4 were found at higher levels in the serum of hepatitis C virus (HCV)-infected patients with HCC than HCV-infected patients with cirrhosis only [42]. Activation of the acute phase response in HCC may be a sign of metabolic reprogramming. Administration of lipopolysaccharide has acute effects on serum cholesterol, triacylglycerides, high-density lipoproteins, and very-low-density lipoproteins [43]. Interestingly, Beigneux et al. demonstrated that stimulation of hepatocytes with lipopolysaccharide reduces PPARα-stimulated acyl-CoA synthetase expression [44]. Tec kinase signaling has an important role in liver regeneration. Protein kinase TEC has been demonstrated to activate ERK within the hepatocyte growth factor signaling pathway [45], while inhibition of MEK/ERK phosphorylation is partly responsible for the anti-tumor activity of the kinase inhibitor sorafenib [46].

Metabolic dysregulation is a common feature of cancer that may involve miR-21-mediated regulation [33,47]. HCC is dependent upon glycolysis rather than mitochondrial oxidation for energy [48]. We found that the miR-21 interactome is predicted to suppress the signaling of hepatic nuclear receptors PPARα/RXRα and LXR/RXR. PPARα activation during fasting stimulates β-oxidation of fatty acids and gluconeogenesis. Feeding reduces PPARα signaling through mTORC1 activation and Akt signaling [49,50]. Meng et al. determined that silencing of PTEN by miR-21 contributes to activation of the Akt pathway in HCC [15]. Additionally, the LXR/RXR pathway activates transcription of lipogenic enzymes in liver as well as their upstream transcription factor SREBP1C in response to ligand binding [51]. However, Wu et al. reported that miR-21 mimic induced SREBP1C in HepG2 cells [47]. Effects of miR-21 on LXR/RXR signaling in primary tumors may be distinct from those in cell culture because of reduced oxygen levels and substrate availability.

We have identified several novel potential miR-21 targets. Among them, PLEKHA1 binds to phosphatidyl inositol (3,4)-diphosphate and interacts with PDZ domains, including PDZ-containing protein tyrosine phosphatase-like protein 1 (PTPL1), a negative regulator of Akt signaling [52,53]. The yeast homolog of *RMND5A*, *GID2*, was originally found to be required for the gluconeogenic to glycolytic metabolic switch that occurs upon replenishment of glucose in a medium containing a nonfermentable carbon source [54]. As a component of the Gid complex, Gid2 is required for polyubiquination of the gluconeogenic enzyme fructose-1,6-bisphosphatase and for degradation of phosphoenolpyruvate carboxykinase [34]. Further investigation is needed to determine whether *RMND5A* mediates the effects of miR-21 on tumor metabolism in a subset of HCC patients.

Our study shows that the expression of three genes, *CAMSAP2*, *DDX1* and *MARCKSL1* with miR-21 binding motifs may mediate decreased survival in HCC patients. The expression of all three of these genes negatively correlated with survival. Dead-box helicase 1 encoded by *DDX1* is an RNA helicase that participates in double-strand break repair [55]. *CAMSAP2* encodes calmodulin-regulated spectrin-associated protein family member 2, a microtubule-associated protein implicated in the regulation of cell motility through microtubule stabilization [56]. The *MARCKSL1* gene encodes MARCKS-related protein, which regulates the actin cytoskeleton and cell migration [57,58]. While miR-21 is predicted to bind the transcript of *MARCKSL1*, its expression positively correlates with miR-21 expression. It is possible that miR-21 suppresses *MARCKSL1* by inhibiting its translation.

Our study is limited to miR-21 targets with known binding motifs for miR-21. In order to fully appreciate the functional role of the miR-21 targetome, it would be necessary to conduct Ago-CLIP and RNAseq analysis on a miR-21 knockout model. A similar analysis was recently performed for the miR-122 targetome in liver [21]. Finally, it would be helpful to validate our findings on the expression of the miR-21 interactome in an independent cohort of patients. 

## 4. Materials and Methods

### 4.1. Argonaute-CLIP Analysis

Ago-CLIP analysis for nine HCC patients and matched benign liver was performed by the Darnell lab as previously reported (GSE97061) [21]. Patient data are given in supplementary Table 1 of Luna et al., 2017 [21]. Ago-CLIP analysis in five mice was previously reported (GSE97058) [21]. This data were accessed through the Gene Expression Omnibus (GEO) data repository. 

### 4.2. Gene Set Enrichment and Pathway Analysis

RNA-seq RPKM data from The Cancer Genome Atlas (cancergenome.nih.gov) were downloaded from firebrowse.org and log_2_ transformed. Differential expression from RNA-seq data of The Cancer Genome Atlas was obtained using the limma package in R cite (cran.r-project.org). Expression data from HCC SK-Hep-1 treated with 20 nmol/L antago-miR-21 and RNA extracted sixteen hours after treatment were previously reported (GSE65892) [33].

Gene set enrichment and pathway analysis were performed using Ingenuity Pathway Analysis (IPA). Log-fold change in RNA-seq abundance in tumor versus normal and associated *p*-value were uploaded to IPA. Pathway analysis was set to consider only human and experimentally observed molecules and/or relationships. Other parameters used the default settings. 

### 4.3. Statistical Analysis

Ago-CLIP enrichment scores for miR-21 binding in HCC were categorized as enriched (logFC > 0) or depleted (logFC < 0). Comparison of categorical data was performed by Chi square. A two-sided Kolmogorov–Smirnov test was used for comparison of density distributions.

Best-fit linear models of quantitative data were produced by least squares regression and significance determined by *t*-test of Pearson’s coefficient. The corr.test() function from psych package in R was used to correct *p*-values of multiple correlations by Holm’s method. Significance was interpreted with α = 0.05 unless otherwise noted.

The OncoLnc package was used for survival analysis (www.oncolnc.org). Cox proportional hazards models were adjusted for age, sex and tumor grade. The significance of the Cox coefficient was corrected for the false-discovery rate as described [59]. 

## Figures and Tables

**Table 1:** Association between miR-21 expression and components of miR-21 interactome dysregulated after anti-sense miR-21 treatment in SK-Hep-1 cells.

Gene Name	Full Name	miR-21 Expression	
		Correlation Coefficient	Adjusted *p*-Value
*ANKRD46*	Ankyrin repeat domain 46	−0.36509	5.30 × 10^−12^
*ARL1*	ADP ribosylation factor like GTPase 1	0.041021	1
*ARRDC3*	Arrestin-domain containing 3	−0.21111	3.96 × 10^−3^
*CAMSAP2*	Calmodulin-regulated spectrin-associated protein family member 2	Not defined *	Not defined *
*CREB3L2*	cAMP responsive element binding protein 3 like 2	0.061419	1
*DDAH1*	Dimethylarginine dimethylaminohydrolase 1	−0.340671	3.32 × 10^−10^
*DDX1*	DEAD-box helicase 1	0.090883	1
*DSTYK*	Dual serine/threonine and tyrosine protein kinase	0.237187	3.07 × 10^−4^
*EGLN1*	Egl-9 family hypoxia inducible factor 1	0.12549213	1
*GBP1*	Guanylate-binding protein 1	−0.0315479	1
*GGCX*	Gamma-glutamyl carboxylase	−0.2833242	1.38 × 10^−6^
*MARCKSL1*	MARCKS like 1	0.27395541	4.52 × 10^−6^
*PAG1*	Phosphoprotein membrane anchor with glycosphingolipid microdomains 1	0.23283137	4.86 × 10^−4^
*PDZD8*	PDZ domain containing 8	−0.0632384	1
*PLEKHA1*	Pleckstrin homology domain containing A1	−0.2021841	8.88 × 10^−3^
*RMND5A*	Required for meiotic nuclear division 5 homolog A	−0.5028372	0
*SLC46A3*	Solute carrier family 46 member 3	−0.4672836	0
*SMARCE1*	SWI/SNF related, matrix associated, actin dependent regulator of chromatin, subfamily e, member 1	0.3145013	1.88 × 10^−8^
*SPG20*	Spastic paraplegia 20 (Troyer syndrome)	−0.2245667	1.11 × 10^−3^
*TBC1D4*	TBC1 domain family member 4	−0.0978456	1
*TGFBR2*	Transforming growth factor β receptor 2	−0.1914894	0.0217
*THBS1*	Thrombospondin 1	−0.1524996	0.391
*TIMP3*	TIMP metallopeptidase inhibitor 3	−0.1720478	0.0994
*TPRG1L*	Tumor protein p63 regulated 1 like	−0.336802	6.19 × 10^−10^

* Gene identifier could not be matched in expression data.

**Table 2:** Expression of the miR-21 interactome and overall survival in 300 HCC patients.

Gene Name	Overall Survival	
	Cox Coefficient	Adjusted *p*-Value
*DDX1*	0.341	0.012
*MARCKSL1*	0.324	0.0171
*CAMSAP2*	0.282	0.0256
*PAG1*	0.214	0.147
*ARRDC3*	0.121	0.408
*DSTYK*	0.109	0.48
*TBC1D4*	0.097	0.543
*SPG20*	0.05	0.802
*SMARCE1*	0.04	0.836
*ARL1*	0.033	0.871
*CREB3L2*	0.017	0.936
*PLEKHA1*	0.013	0.954
*DDAH1*	−0.006	0.976
*EGLN1*	−0.021	0.918
*TPRG1L*	−0.037	0.866
*THBS1*	−0.042	0.836
*GBP1*	−0.069	0.687
*PDZD8*	−0.075	0.645
*GGCX*	−0.099	0.514
*ANKRD46*	−0.11	0.505
*RMND5A*	−0.104	0.493
*TGFBR2*	−0.136	0.365
*TIMP3*	−0.212	0.135
*SLC46A3*	−0.214	0.151

## Supplementary Materials

Supplementary materials can be found at http://www.mdpi.com/1422-0067/19/3/851/s1.

## Abbreviations

Ago-CLIP-seq	High-throughput sequencing of RNAs isolated by cross-linking and immunoprecipitation of Argonaute
HCC	Hepatocellular carcinoma
TCGA-LIHC	The cancer genome atlas liver hepatocellular carcinoma
3′UTR	3′ untranslated region
5′UTR	5′ untranslated region
CDS	Coding DNA sequence
IPA	Ingenuity Pathway Analysis
PDCD4	Programmed cell death protein 4
PTEN	Phosphatase and tensin homolog
RECK	Reversion-inducing-cysteine-rich protein with kazal motifs
TIMP3	Metalloproteinase inhibitor 3
LXR/RXR	Liver X receptor/retinoid X receptor
PPARα/RXRα	Peroxisome proliferator-activated receptor alpha/retinoid X receptor alpha
HCV	Hepatitis C virus
RNASeq	RNA sequencing
