# Supplementary material for: A Comprehensive Analysis of Argonaute-CLIP Data Identifies Novel, Conserved and Species-Specific Targets of miR-21 in Human Liver and Hepatocellular Carcinoma
Source: Int J Mol Sci. 2018 Mar 14;19(3):851. doi: 10.3390/ijms19030851 (PMC5877712; doi:10.3390/ijms19030851)
Supplement: Supplementary file 1 [file ijms-19-00851-s001.pdf]

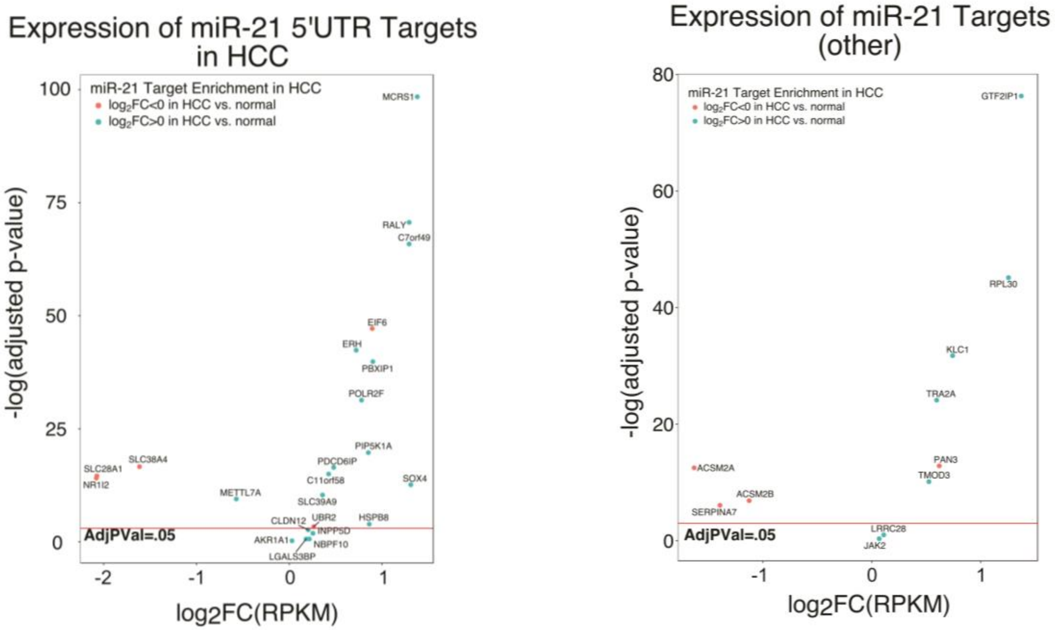

(**a**) (**b**)

Figure S1. Expression of miR-21 targets not in CDS or 3’UTR. Volcano plot of gene expression for miR-21 5’UTR targets (**a**). Volano plot of gene expression for miR-21 targets in introns, deep intergenic regions or downstream 10K (**b**).

Table S1. miR-21 binding sites in human and mouse homologs for 24 candidate miR-21 targets.

Gene Name	Full name	Number of miR-21 binding sites in mouse homolog	Number of miR-21 binding sites	Location of binding sites
ANKRD46	ankyrin repeat domain 46	0	1	3′UTR
ARL1	ADP ribosylation factor like GTPase 1	0	1	3′UTR
ARRDC3	Arrestin-domain containing 3	1	0	5′UTR
		0	1	CDS
		0	1	3′UTR
CAMSAP2	Calmodulin-regulated spectrin-associated protein family member 2	0	1	CDS
CREB3L2	cAMP responsive element binding protein 3 like 2	1	1	CDS
DDAH1	Dimethylarginine dimethylaminohydrolase 1	0	1	3′UTR
DDX1	DEAD-box helicase 1	1	1	CDS
DSTYK	Dual serine/threonine and tyrosine protein kinase	0	1	CDS
EGLN1	Egl-9 family hypoxia inducible factor 1	0	1	3′UTR
GBP1	Guanylate-binding protein 1	0	1	3′UTR
GGCX	Gamma-glutamyl carboxylase	0	1	CDS
MARCKSL1	MARCKS like 1	1	1	3′UTR
PAG1	Phosphoprotein membrane anchor with glycosphingolipid microdomains 1	1	1	3′UTR
PDZD8	PDZ domain containing 8	0	1	CDS

Gene Name	Full name	Number of miR-21 binding sites in mouse homolog	Number of miR-21 binding sites	Location of binding sites
		1	0	3′UTR
PLEKHA1	Pleckstrin homology domain containing A1	0	2	CDS
		1	0	3′UTR
RMND5A	Required for meiotic nuclear division 5 homolog A	1	1	CDS
		2	0	3′UTR
SLC46A3	Solute carrier family 46 member 3	0	1	3′UTR
SMARCE1	SWI/SNF related, matrix associated, actin dependent regulator of chromatin, subfamily e, member 1	0	1	3′UTR
SPG20	Spastic paraplegia 20 (Troyer syndrome)	0	1	3′UTR
TBC1D4	TBC1 domain family member 4	0	1	3′UTR
TGFBR2	Transforming growth factor beta receptor 2	1	1	3′UTR
THBS1	Thrombospondin 1	0	1	3′UTR
TIMP3	TIMP metallopeptidase inhibitor 3	0	2	CDS
		0	1	3′UTR
TPRG1L	Tumor protein p63 regulated 1 like	1	1	3′UTR

Table S2. Protein function and canonical pathway involvement for 24 candidate miR-21 targets. Data from Ingenuity KnowledgeBase.

Gene Name	Full name	Protein Function/Functional Domains	Canonical Pathways
ANKRD46	ankyrin repeat domain 46	Transcription regulator	-
ARL1	ADP ribosylation factor like GTPase 1	Enzyme, enzyme activator activity, GTPase, GTP binding, GTP binding domain-switch II region, protein domain specific binding	-
ARRDC3	Arrestin-domain containing 3	G-protein-coupled receptor binding, PPXY motif, protein binding	-
CAMSAP2	Calmodulin-regulated spectrin-associated protein family member 2	Microtubule binding, protein binding	-
CREB3L2	cAMP responsive element binding protein 3 like 2	DNA binding, double-stranded DNA binding, nucleic acid binding, RNA polymerase II core promoter proximal region sequence-specific binding transcriptional activator activity, sequence-specific DNA binding, transcription regulator	-
DDAH1	Dimethylarginine dimethylaminohydrolase 1	Amidinotransferase, amino acid binding, dimethylarginase, enzyme, zinc ion binding	-
DDX1	DEAD-box helicase 1	ATP-dependent helicase, chromatin binding, double-stranded RNA binding, enzyme, helicase, nuclease, poly(A)-binding protein, protein binding, RNA binding, RNA helicase, transcription cofactor	-
DSTYK	Dual serine/threonine and tyrosine protein kinase	Kinase, protein threonine/tyrosine kinase	-
EGLN1	Egl-9 family hypoxia inducible factor 1	2-oxoglurarate:oxygen oxidoreductase, enzyme, enzyme binding, protein binding	HIF1α signaling
GBP1	Guanylate-binding protein 1	Actin binding, cytokine binding, enzyme, enzyme binding, GDP binding, GMP binding, GTPase, GTPase domain, GTP binding, heat shock protein binding, helical domain, identical protein binding, protein binding, protein homodimerization, spectrin binding	--
GGCX	Gamma-glutamyl carboxylase	Enzyme, gamma-glutamyl carboxylase	-
MARCKSL1	MARCKS like 1	Effector domain, protein binding	-
PAG1	Phosphoprotein membrane anchor with glycosphingolipid microdomains 1	Csk binding site, cytoplasmic domain, extracellular domain, membrane-association domain, palmitoylation site, protein binding, SH2-domain binding, transmembrane domain, transmembrane receptor protein tyrosine kinase adaptor protein	B cell receptor signaling, T cell receptor signaling
PDZD8	PDZ domain containing 8	Coiled-coil domain	-

Gene Name	Full name	Protein Function/Functional Domains	Canonical Pathways
PLEKHA1	Pleckstrin homology domain containing A1	PDZ-domain binding, phosphatidylinositol binding, protein binding	iCOS-iCOSL Signaling in T helper Cells; PI3K Signaling in B lymphocytes
RMND5A	Required for meiottic nuclear division 5 homolog A	Protein binding	--
SLC46A3	Solute carrier family 46 member 3	-	-
SMARCE1	SWI/SNF related, matrix associated, actin dependent regulator of chromatin, subfamily e, member 1	Acidic domain, chromatin binding, coiled-coil domain, ligand-dependent nuclear receptor interactor, N-acetyltransferase, NHRLI domain, nuclear localization sequence, proline-rich domain, protein binding, protein N-terminus binding, RNA binding, transcription regulator	AMPK signaling, glucocorticoid receptor signaling, hereditary breast cancer signaling, RAR activation, role of BRCA1 in DNA damage response
SPG20	Spastic paraplegia 20 (Troyer syndrome)	MIT domain, protein binding, ubiquitin protein ligase binding	-
TBC1D4	TBC1 domain family member 4	Phosphotyrosine-binding domain, protein binding	-
TGFBR2	Transforming growth factor beta receptor 2	Cytoplasmic domain, cytoplasmic kinase domain, cytosolic tail domain, extracellular domain, glycosaminoglycan binding, intracellular domain, kinase, kinase domain membrane-proximal domain, N-linked glycosylation site, protein binding, protein heterodimerization, protein homodimerization, protein kinase binding, protein serine/threonine kinase, serine/threonine kinase domain, SMAD binding, tail domain, TGFbeta binding, transforming growth factor-beta receptor, transforming growth factor-beta receptor binding, transmembrane domain, transmembrane receptor, transmembrane receptor protein serine/threonine kinase, type II transforming growth factor-beta receptor	Antiproliferative role of TOB in T cell signaling, cardiac hypertrophy signaling, chronic myeloid leukemia signaling, colorectal cancer metastasis signaling, epithelial adherens junction signaling, factors promoting cardiogenesis in vertebrates, germ cell-Sertoli cell junction signaling, glucocorticoid receptor signaling, hepatic fibrosis/stellate cell activation, human embryonic stem cell pluripotency, inhibition of angiogenesis by TSP1, molecular mechanisms of cancer, NF-κB signaling, osteoarthritis pathway, p38 MAPK signaling, pancreatic adenocarcinoma signaling, PPARα/RXRα activation, protein kinase A signaling, PTEN signaling, regulation of IL-2 expression in activated and anergic T lymphocytes, regulation of the epithelial-mesenchymal transition pathway, role of NFAT in cardiac hypertrophy, STAT3 pathway, TGF-β

Gene Name	Full name	Protein Function/Functional Domains	Canonical Pathways
			signaling, Th1 and Th2 activation pathway, Th2 pathway, T helper cell differentiation, tight junction signaling, Wnt/β-catenin signaling
THBS1	Thrombospondin 1	Alpha3 beta1 integrin-binding domain, binding protein, calcium binding loop, CD36-binding domain, cell-binding domain, collagen binding, EGF-like domain, extracellular matrix binding, fibroblast growth factor binding, fibronectin binding, globular domain, glycoprotein binding, heparin binding, heparin binding domain, IAP binding domain, integrin binding, laminin binding, L-lectin like domain, low-density lipoprotein binding, oligomerization domain, phospholipid binding, procollagen-like segment, protein binding, protein complex binding, signal peptide, stalk region, TGFbeta binding, thrombospondin-1 motif, thrombospondin properdin-like repeat, thrombospondin type 1 repeat, thrombospondin type 2 repeat, thrombospondin type 1 repeat, thrombospondin type 2 repeat, thrombospondin type 3 repeat	Bladder cancer signaling, inhibition of angiogenesis by TSP1, p53 signaling
TIMP3	TIMP metallopeptidase inhibitor 3	Metalloendopeptidase inhibitor, protein binding	Glioma invasiveness signaling, inhibition of matrix metalloproteases, leukocyte extravasation signaling, oncostatin M signaling, osteoarthritis pathway
TPRG1L	Tumor protein p63 regulated 1 like	Identical protein binding, protein binding	--
